# Continuous non-contact vital sign monitoring in neonatal intensive care unit

**DOI:** 10.1049/htl.2014.0077

**Published:** 2014-09-23

**Authors:** Mauricio Villarroel, Alessandro Guazzi, João Jorge, Sara Davis, Peter Watkinson, Gabrielle Green, Asha Shenvi, Kenny McCormick, Lionel Tarassenko

**Affiliations:** 1 Institute of Biomedical Engineering, Department of Engineering Science, University of Oxford, Oxford, UK; 2 Neonatal Unit, John Radcliffe Hospital, Oxford University Hospitals Trust, UK; 3 Nuffield Department of Clinical Neurosciences, University of Oxford, Oxford, UK

**Keywords:** paediatrics, biomedical optical imaging, patient monitoring, patient care, medical image processing, pneumodynamics, oxygen, oximetry, biochemistry, cardiology, blood vessels, estimation theory, video recording, video cameras, biomedical equipment, feature extraction, continuous noncontact vital sign monitoring, neonatal intensive care unit, continuous vital sign monitoring technologies, continuous pre-term infant monitoring, hospital, adhesive electrodes, adhesive sensors, direct patient contact, infant stress, infant pain, infant skin damage, superficial blood vessel colour change measurement, superficial blood vessel volume change measurement, cardiac cycle, digital video camera, ambient light, continuous heart rate estimation, breathing rate estimation, adult healthy human volunteers, clinical letter, high-dependency care area, NICU, video-based noncontact monitoring methods, continuous respiratory rate estimation, continuous oxygen saturation estimation, infant nursing, incubators, stable sections, minimal infant motion, mean absolute error, camera-derived estimates, reference heart rate value, electrocardiogram, ECG-derived value, pulse oximeter, bradycardia identification, major desaturation, video signal processing algorithms, time 4 day, O_2_

## Abstract

Current technologies to allow continuous monitoring of vital signs in pre-term infants in the hospital require adhesive electrodes or sensors to be in direct contact with the patient. These can cause stress, pain, and also damage the fragile skin of the infants. It has been established previously that the colour and volume changes in superficial blood vessels during the cardiac cycle can be measured using a digital video camera and ambient light, making it possible to obtain estimates of heart rate or breathing rate. Most of the papers in the literature on non-contact vital sign monitoring report results on adult healthy human volunteers in controlled environments for short periods of time. The authors' current clinical study involves the continuous monitoring of pre-term infants, for at least four consecutive days each, in the high-dependency care area of the Neonatal Intensive Care Unit (NICU) at the John Radcliffe Hospital in Oxford. The authors have further developed their video-based, non-contact monitoring methods to obtain continuous estimates of heart rate, respiratory rate and oxygen saturation for infants nursed in incubators. In this Letter, it is shown that continuous estimates of these three parameters can be computed with an accuracy which is clinically useful. During stable sections with minimal infant motion, the mean absolute error between the camera-derived estimates of heart rate and the reference value derived from the ECG is similar to the mean absolute error between the ECG-derived value and the heart rate value from a pulse oximeter. Continuous non-contact vital sign monitoring in the NICU using ambient light is feasible, and the authors have shown that clinically important events such as a bradycardia accompanied by a major desaturation can be identified with their algorithms for processing the video signal.

## 1 Introduction

The technology for monitoring infants admitted to the Neonatal Intensive Care Unit (NICU) has hardly changed in the past 30 years, since the advent of pulse oximetry [1]. Furthermore, the current methods for monitoring heart rate, respiratory rate and oxygen saturation require the use of adhesive electrodes or sensors. These can damage the fragile skin of pre-term infants, and cause stress and pain. In this Letter, we report the preliminary results from a video-based, non-contact monitoring method which does not require any electrodes or sensors to be attached to the infant, just a measurement of the ambient light reflected from the infant's skin by a digital video camera positioned securely over the incubator.

Camera-based non-contact estimation of heart rate in the NICU using standard video cameras was first reported in 2012 [2]. Seven infants were monitored in this study, each for a maximum of 30 s with a webcam 20 cm away from the face and with special illumination.

A team of academic researchers working with Philips (Eindhoven, The Netherlands) have since made video camera recordings of 19 infants in two NICUs in California and the Netherlands [3]. The camera was placed on a tripod at approximately 1 m from the infant. The infants were monitored for periods of up to 5 min. Regions of interest from the head, arms or thorax were manually selected from the images. The heart rate was estimated from Fast Fourier transform (FFT) analysis of the green channel of the RGB camera. In 13 out of 19 infants, it was possible to derive heart rate estimates for 90% of the time. No attempt was made to estimate any other vital sign.

Another group of researchers have recently reported spot measurements of heart rate using a Sony camera, and of respiratory rate using an infra-red thermal camera [4]. Both the cameras were mounted on a stable tripod, next to an open incubator. Images were recorded on one occasion from six infants and on two occasions from another infant. Open-source code developed by the Freeman group at MIT [5] was used to amplify the colour variations in the Sony camera images and visualise the pulse. Respiratory rate was estimated using the software supplied by the manufacturer of the thermal camera, based on tracking the changes in temperature around the baby's nostrils during the breathing cycle.

In 2011, Abbas *et al.* [6] described methods to derive respiratory rate from an infrared thermal camera. They monitored seven premature infants with a median gestational age of 29 weeks receiving respiratory support via continuous positive airway pressure (CPAP) in the NICU. Video data from the infants were recorded for 6 min. Respiratory rate was estimated using a continuous wavelet transform of the temperature difference between the inspiration and expiration phases, with a camera positioned ∼ 0.5 cm from the incubator. These authors later expanded their methodology to enable them to track several geometric regions of interest over the neonate's body [7].

The use of standard cameras and thermal imaging is further explored in [8]. A camera with a charge-coupled device (CCD) sensor was used to derive heart rate from small regions of interest (typically 10 × 10 pixels). By analysing the temperature distribution in the nasal region from an infrared camera, respiratory rate was derived in 10-min recordings from seven pre-term infants in the NICU.

It is important for the management of infants in the NICU that vital sign measurement is continuous. In this Letter, we show how the cardiorespiratory status of an infant can be tracked from continuous measurements of heart rate, respiratory rate and peripheral oxygen saturation (*S_p_*O_2_) derived from an analysis of the reflectance signals acquired with a digital video camera in a real hospital environment, with regular ambient light and without affecting patient care.

## 2 Methodology

We have previously described [9] our algorithms for deriving estimates of heart rate, respiratory rate and changes in oxygen saturation by processing the RGB signals recorded by a digital video camera typically positioned 1 m away from the subject. In our initial clinical study, the subjects were adult patients undergoing haemodialysis at the Oxford Kidney Unit [9]. We have now begun a clinical study with 30 pre-term infants (REC reference number: 13/SC/0597) in the high-dependency care area of the NICU at the John Radcliffe Hospital in Oxford. Each pre-term infant is double monitored, using both the existing Philips IntelliVue MX800 Patient Monitor, which is the standard of care in the Oxford NICU, and our digital video camera, during the daytime for at least four consecutive days.

**Figure 1:**
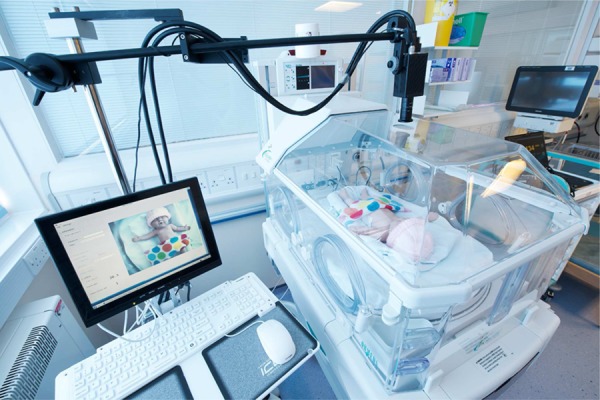
Monitoring equipment showing a mannequin inside the study incubator in the Oxford NICU Digital video camera and camera arm are clearly visible

To monitor the vital signs of these pre-term infants continuously using the video camera, without affecting patient care, we modified an incubator as shown in Fig. 1. A hole with a 3 cm diameter was cut in the top of the incubator canopy. To comply with hospital health and safety regulations, we performed a continuous test over 2 weeks of the temperature and humidity within the modified, empty incubator. No significant differences were found with respect to the same incubator prior to the modifications, and so our modifications were approved by the Medical Research Ethics Committee (MREC).

The camera is mounted at the end of a specially designed arm, to minimise disturbance to the care of the infant. The videos are captured with a resolution of 1620 × 1236 pixels, with 8 bits per pixel, at 20 frames per second. The video camera (JAI AT-200CL digital 3CCD progressive scan) has three separate CCD sensors to measure red, green and blue light intensity independently. We developed our own software for implementing image acquisition and processing on a Xilinx Spartan Field Programmable Gate Array (FPGA) board, using a workstation running the Fedora Linux Operating System. To comply with the requirements of our MREC approval and to preserve patient privacy, the images are stored using a customised binary format and saved in an encrypted storage cluster.

**Figure 2:**
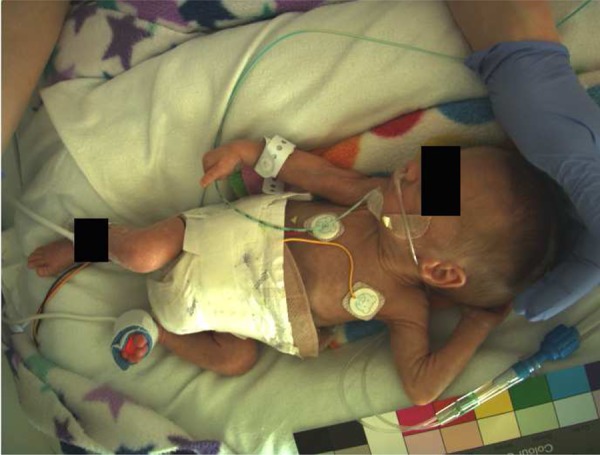
Image from video camera of infant resting quietly in the incubator

Fig. 2 shows an infant, born at 28 weeks gestation, at 31 weeks corrected gestational age and weighing 1200 g, who was monitored for four consecutive days during his stay in the NICU.

One of the main goals of our study is minimal interference with regular patient care. There are therefore periods during which the video data cannot be analysed. These correspond to the following occurrences:

- Regular interaction between the clinical staff and the baby;
- Clinical interventions such as heel pricks, used to withdraw blood for lab tests;
- Baby taken out of the incubator to be held by the mother (kangaroo care).

We label the stable periods of video data outside these occurrences as ‘valid camera data’. To identify these stable periods, we use a non-parametric statistical background image estimation algorithm based on the methods described in [10] to classify the video frames into ‘foreground’ (visible skin areas) and ‘background’ pixel collections. As the videos are recorded under variable lighting conditions, the background model is updated by giving greater weight to newer observations.

Once stable periods have been identified, two regions of interest (ROI) are computed: the subject ROI, ROI_S_, a rectangular area on the patient's skin, such as the face, head or neck, for which the changes in colour and volume with every heart beat can be estimated from analysis of the video signal and the background ROI, ROI_R_, from which the artefacts associated with strong light sources can be minimised, as described in [9].

**Figure 3:**
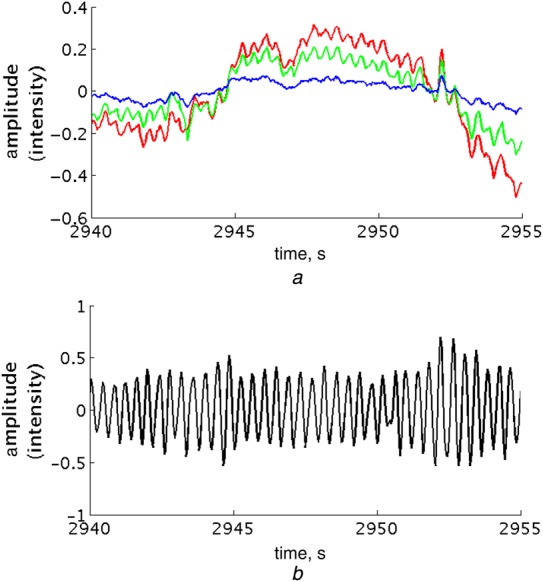
Region of interest extraction for a 15-second video section *a* Mean of the light intensity of pixels from the red, green and blue channels of a 150 × 150 region of interest *b* Reflectance PPG signal derived from ICA of the three channels

The vital sign estimation algorithms start with the extraction of the mean intensity for the pixels from the three channels of ROI_S_, typically within a 150 × 150 ROI. We use independent component analysis (ICA) [11] of these three channels to obtain the reflectance photoplethysmogram (PPG) waveform, which has a strong cardiac-synchronous component, as shown in Fig. 3.

We model the output of the ICA process with a set of auto-regressive (AR) models [9]. AR modelling allows us to identify regular frequencies in the low-amplitude, noisy reflectance PPG signal recorded by the video camera. The dominant frequency in the signal is the cardiac frequency, as a result of the changes in colour and volume of superficial vessels with each heart beat. To capture the heart rate range of a typical pre-term infant, we use a finite-impulse response (FIR) band-pass filter (BPF1) with cut-off frequencies of 1.3 and 5 Hz, corresponding to heart rates of 78 and 300 beats per minute.

Breathing-synchronous motion also modulates the amplitude of the reflectance PPG signal recorded by the video camera; hence, there is also a respiratory peak in the spectrum, but of much lower amplitude than the cardiac peak. The reflectance signal is filtered independently with an FIR band-pass filter (BPF2) tuned to the expected frequency range of respiratory rates in pre-term infants (0.33–1.67 Hz, corresponding to 20–100 breaths per minute). This filtering approach to enhance the respiratory rate information is described in more detail in [9], with the difference that the frequency cut-offs of BPF2 are much higher here, as pre-term infants breathe much faster than adults.

The cardiac and respiratory frequencies are enhanced using band-pass filters BPF1 and BPF2 applied to 8-second and 20-second windows, respectively, prior to modelling with standard low-order AR models [9].

For oxygen saturation estimation, the sliding windows of the heart rate estimation algorithm (moved 1 s at a time) are analysed. As a validation step for now, only those windows for which the camera-derived heart rate estimates agree with the reference measurements (within ±2 beats/minute) are used to estimate oxygen saturation from the outputs of the red and blue channels, each filtered with BPF1.

## 3 Results

We report the results from the first two of the infants monitored in our study for nearly 40 h in total. The recordings were made during the months of April and May, and data collection will continue until the end of the calendar year. As one of the main goals of our study is not to interfere with regular patient care, the video recordings are made within the unmodified environment of the NICU, which is a mixture of natural lighting and artificial lighting (mostly fluorescent). Changes in artificial lighting can affect the estimation of the vital sign values. We observed an aliased mains frequency peak at 10 Hz. As explained in our previous paper [9], pole cancellation in AR models allows us to cope with the interference from such aliased frequencies.

### 3.1 Measurements with respect to conventional monitoring

There are two sources of heart rate information available from the Philips monitor: a measurement derived from the beat-to-beat intervals between successive *R*-peaks in the electrocardiogram (ECG) and one derived from the beat-to-beat intervals between successive peaks in the contact PPG waveform (red channel from the pulse oximeter). After synchronising these two data streams, we investigated the agreement between the two heart rate estimates and found that 94% of the ECG-derived estimates and PPG-derived estimates were within ±4 beats per minute of each other. This gives us a measure of the error which we should aim for when comparing our camera-derived estimates of heart rate with the ECG-derived heart rate.

The situation is more complicated with respiratory rate. The electrical impedance pneumography (EIP) signal, obtained by injecting a high-frequency AC current between the pair of ECG electrodes across the chest, is notoriously noisy. In an attempt to validate the estimates computed by the Philips patient monitor, we acquired the EIP waveform and band-pass filtered it using a 10 second-long FIR filter with frequency cut-offs equivalent to 20 and 100 breaths per minute, the limits of the typical range of neonatal respiratory rates. Re-derived estimates of respiratory rate were then calculated by finding the peak in the power spectrum of 20 s sliding windows of this signal, updated every 2 s. (A 1024-point FFT with Hamming windowing was employed to estimate the power spectrum.) We found, on a typical 7-hour record, that just under 60% of the values for the IntelliVue MX800 monitor and the corresponding re-derived estimates were within ±10 breaths per minute of each other. In subsequent analysis (e.g. in that used to derive Fig. 3*b*), we compare the camera-derived respiratory rate estimates with this re-derived reference.

An accurate calculation of the amplitude of the pulsatile reflectance PPG signal (AC value) at two wavelengths is much more difficult than for the case of the video signals recorded in the adult study in the Renal Unit [9], mainly because the heart rate of pre-term infants is more than double that of the adult patients in that study. Therefore, we instead computed the DC values at the two wavelengths (red and blue) previously used [9], and the results reported below show that changes in oxygenation during an apnoeic episode may be followed using the changes in the values of the DC ratio (red/blue).

### 3.2 Continuous, non-contact monitoring

We next investigated the lengths of time for which we were able to monitor the infants continuously. Each infant was monitored on four consecutive days, for periods ranging from 4.14 to 7.32 h (total of 39.8 h), apart from day 2 for Patient 1 (when technical issues prevented a lengthier recording). Table 1 summarises the periods of continuous monitoring, as determined from retrospective, off-line analysis, which is now explained below.

**Table 1:** Summary of each vital-sign monitoring session for two infants, each monitored on four consecutive days during the daytime

Patient	Session	Total recording time, h	Valid camera data		Continuous heart rate estimation				
			Time, h	Time, %	Time, h	Time, % valid data	Time, % all data	RMSE, bpm	MAE, bpm
patient 1	day 1	5.70	4.32	77.4	3.83	86.7	67.2	3.87	2.67
	day 2	0.89	0.83	93.5	0.62	74.4	69.6	3.45	2.23
	day 3	4.55	3.22	70.8	2.81	87.2	61.8	3.78	2.56
	day 4	5.25	2.71	51.6	2.23	82.3	42.5	4.09	3.09
patient 2	day 1	4.14	2.78	67.0	2.03	73.1	49.0	3.92	2.91
	day 2	7.32	3.34	45.6	2.81	84.2	38.4	4.27	3.14
	day 3	6.99	4.61	65.9	3.44	74.6	49.2	4.15	3.06
	day 4	4.90	2.94	60.1	2.35	79.8	48.0	4.04	2.97
total		39.8	24.9		20.1				
average				66.5		80.3	53.2%	3.95	2.83

Valid camera data, during which the infant is resting quietly inside the incubator, was available for 66.5% of the time (24.9 h). For 80.3% of the valid-data time (20.1 h), accurate estimates of heart rate are derived on a continuous basis, using 8-second sliding windows. The RMSE and MAE are derived with respect to the ECG-derived heart rate from the Philips IntelliVue MX800 Patient Monitor.

The ROIs used to derive the results are manually segmented from review of the beginning of the video recording and then automatically tracked as the baby moves [10]. As shown in Table 1, the ‘stable periods’ during which ‘valid camera data’ are available amount to 66.5% of the total recording time (24.9 h). For 33.5% of the time, the infant was out of the cot (kangaroo care) or the cot was covered up during a period of quiet time or a clinical procedure was being carried out.

However, even during stable periods, we found other phenomena that prevented us from estimating the vital sign values accurately. These include:

- Major changes in lighting conditions in the NICU.
- Variation in the baby's activity patterns. Small pre-term infants make irregular movements throughout the day, both during wakefulness and sleep, and these make it difficult to compute the frequency components of the heart rate and respiratory rate in the pulsatile reflectance waveform. These motion or activity patterns are not fully understood and we are planning to study them in greater detail, as they may be indicators of the baby's well-being.
- The lack of visible skin area. Although Fig. 2 represents a typical scenario, there are also times during which the baby is covered and not much skin area is visible, making parameter estimation challenging.

The above three factors combine to reduce the time for which we could estimate vital sign values accurately from 24.9 to 20.1 h (i.e. for 80.3% of the valid camera data). For those 20.1 h, we were able to obtain accurate estimates of heart rate continuously, using 8 s sliding windows. Estimates were deemed to be accurate if the root-mean square error (RMSE) between the camera estimate and the ECG-derived value (from the Intellivue MX800 monitor) was less than 5 beats/minute. Overall, for the data labelled as accurate according to this criterion, the RMSE with respect to the ECG-derived heart rate varied from 3.45 to 4.27 beats per minute for the 8 sessions (mean = 3.95 beats per minute). The mean absolute error (MAE) for the same 8 s windows varied from 2.23 to 3.14 beats per minute for the 8 sessions (mean = 2.83 beats per minute).

### 3.3 Bradycardic episode

**Figure 4:**
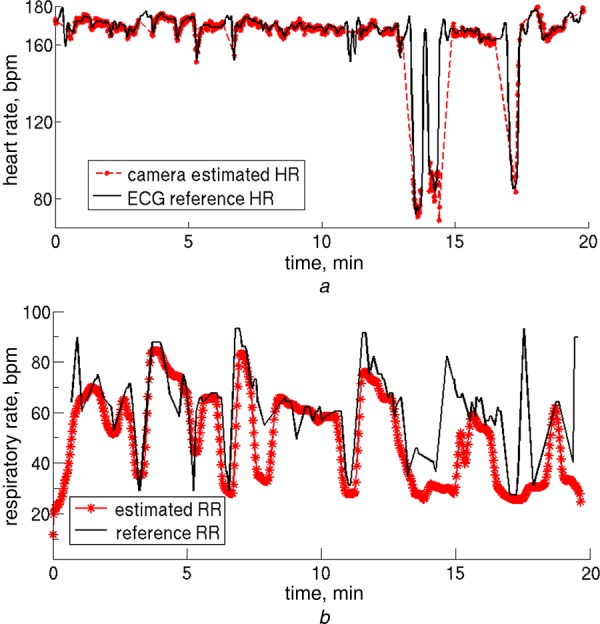
Estimates for heart rate and respiratory rate: *a* Heart rate estimates derived from the reflectance PPG signal extracted from the three colour channels of the video camera using ICA are shown in red; the ECG-derived estimates from the Philips monitor (reference values) are shown in black *b* Respiratory rate estimates derived from the same sections of video camera data are shown in red; the EIP-derived estimates (reference values) are shown in black

**Figure 5:**
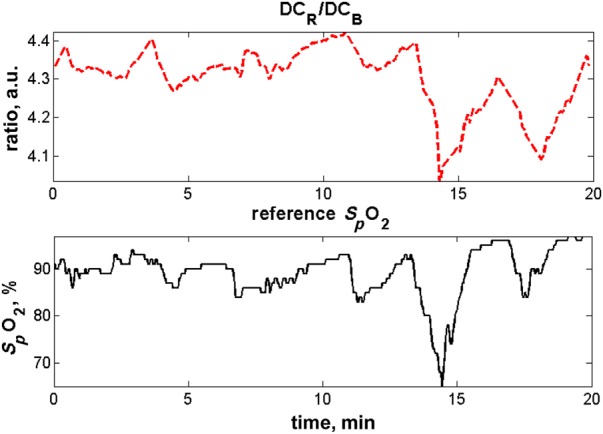
Estimates of oxygen saturation and pulse-oximeter estimates Estimates of oxygen saturation derived from the red and blue channels of the video camera are shown in red; the pulse-oximeter estimates from the Philips monitor (reference values) are shown in black DC_R_/DC_B_ represents the ratio of the DC values in the red and blue channels

Episodes of bradycardia are a frequent occurrence in the pre-term infant, as a result of the immaturity of the cardiorespiratory system. They can also be caused by neonatal sepsis. We analysed a typical 20 min section of data, during which there is a clear bradycardia (decrease of heart rate from 170 to 70 beats per minute) around *t* = 14 min (see Figs. 4 and 5).

The plots of heart rate, respiratory rate and oxygen saturation, both the reference values from the Philips monitor (in black) and the video camera derived estimates (in red) are shown in Figs. 4 and 5. During the 20-minute period shown in the plots, there are huge swings in respiratory rate (from 20 to 80 breaths per minute), identified both by conventional monitoring and our processing of the camera signals. The bradycardia is linked to a major desaturation down to an *S_p_*O_2_ value of 70%, which is tracked by the changes in the value of the DC ratio (red/blue).

**Figure 6:**
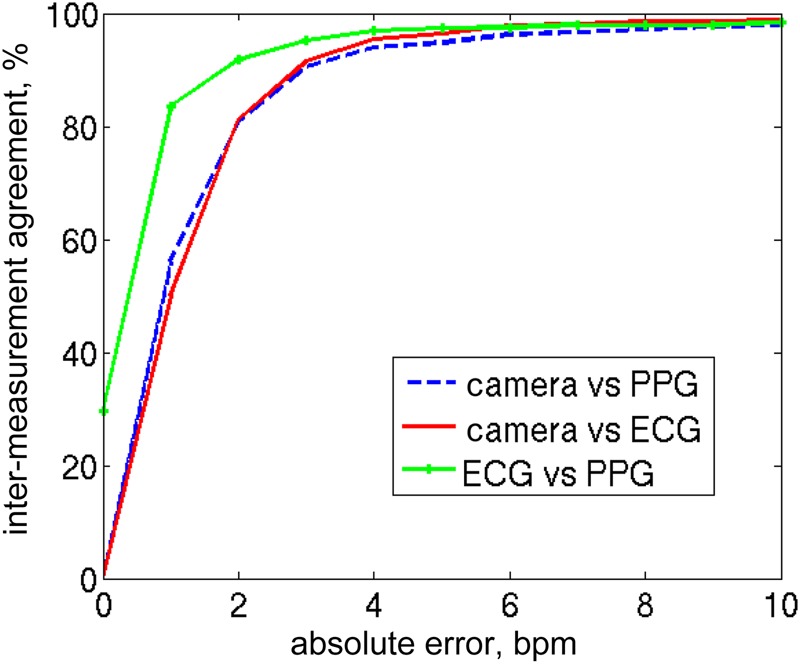
Inter-method agreement (percentage of estimates within a given number of beats/minute) for pairwise comparison of the three methods of deriving heart rate estimates during the 20 min segment analysed in Figs. 4 and 5

Fig. 6 shows a plot of pairwise comparisons between the three available methods for estimating heart rate, during this 20 min period of monitoring: ECG against pulse oximeter, camera-derived against ECG and camera-derived against pulse oximeter.

It shows that 91.9% of the ECG and pulse oximeter values are within 2 beats/min of each other. The equivalent figure for the camera-derived values and the ECG or pulse oximeter values is 81.2%. If an absolute error of 4 beats/min is acceptable, the accuracy of camera-derived estimates is not significantly worse than those obtained with conventional monitoring, as demonstrated by the convergence of the three traces on Fig. 6 for absolute errors of 4 beats/min and above. For a typical neonatal heart rate of 150 beats/min, an error of 4 beats/min corresponds to a 2.7% difference. Even during episodes of bradycardia (with heart rates of about 75 beats/min), a difference of 5% is not clinically significant.

The changes in oxygen saturation during the bradycardic episode are clinically important. The oxygen saturation that is estimated by calculating the ratio of the DC values of the camera PPG signal at the red and blue wavelengths is that of the blood present in the skin tissue of the pre-term infant being monitored. Tissue oxygen saturation is believed to be correlated with central arterial oxygen saturation and therefore with peripheral arterial oxygen saturation as measured with pulse oximeters [12]. However, as we compute DC ratios rather than AC ratios as in pulse oximetry, the results depend on venous as well as arterial oxygen saturation, but this is not a significant concern for the case of a rapid desaturation such as here. This demonstrates that the oxygen saturation signal is present in video segments recorded during stable periods, and that this signal tracks oxygen saturation decreases associated with neonatal apnoeas over an extended period of time, which is a novel and clinically useful result.

## 4 Conclusions

We have demonstrated that it is possible to monitor heart rate, respiratory rate and changes in oxygen saturation continuously in the NICU, with an accuracy which is clinically useful. To the best of our knowledge, this is the first study establishing the feasibility of continuous non-contact monitoring of cardiorespiratory vital signs in hospital using a digital video camera and regular ambient light, for several hours, without affecting patient care. We have now successfully monitored fifteen infants continuously during the daytime. We also believe that this is the first report of non-contact monitoring of oxygen saturation in the NICU using ambient light and a single video camera. We have shown that our algorithms for processing the camera reflectance signals are capable of identifying clinically important events such as a bradycardia accompanied by major desaturation.
